# Genome-Wide Association Study Identifies *GPC5* as a Novel Genetic Locus Protective against Sudden Cardiac Arrest

**DOI:** 10.1371/journal.pone.0009879

**Published:** 2010-03-25

**Authors:** Dan E. Arking, Kyndaron Reinier, Wendy Post, Jonathan Jui, Gina Hilton, Ashley O'Connor, Ronald J. Prineas, Eric Boerwinkle, Bruce M. Psaty, Gordon F. Tomaselli, Thomas Rea, Nona Sotoodehnia, David S. Siscovick, Gregory L. Burke, Eduardo Marban, Peter M. Spooner, Aravinda Chakravarti, Sumeet S. Chugh

**Affiliations:** 1 Heart Institute, Cedars-Sinai Medical Center, Los Angeles, California, United States of America; 2 Center for Complex Diseases Genomics, McKusick-Nathans Institute of Genetic Medicine, the Johns Hopkins University School of Medicine, Baltimore, Maryland, United States of America; 3 Division of Cardiology, the Johns Hopkins University School of Medicine, Baltimore, Maryland, United States of America; 4 Department of Emergency Medicine, Oregon Health and Science University, Portland, Oregon, United States of America; 5 Department of Public Health Sciences, Wake Forest University, Winston-Salem, North Carolina, United States of America; 6 School of Public Health, University of Texas Health Science Center, Houston, Texas, United States of America; 7 Division of Cardiology, Department of Medicine, University of Washington, Seattle, Washington, United States of America; 8 Group Health Research Institute, Group Health, Seattle, Washington, United States of America; Innsbruck Medical University, Austria

## Abstract

**Background:**

Existing studies indicate a significant genetic component for sudden cardiac arrest (SCA) and genome-wide association studies (GWAS) provide an unbiased approach for identification of novel genes. We performed a GWAS to identify genetic determinants of SCA.

**Methodology/Principal Findings:**

We used a case-control design within the ongoing Oregon Sudden Unexpected Death Study (Oregon-SUDS). Cases (n = 424) were SCAs with coronary artery disease (CAD) among residents of Portland, OR (2002–07, population ∼1,000,000) and controls (n = 226) were residents with CAD, but no history of SCA. All subjects were of White-European ancestry and GWAS was performed using Affymetrix 500K/5.0 and 6.0 arrays. High signal markers were genotyped in SCA cases (n = 521) identified from the Atherosclerosis Risk in Communities Study (ARIC) and the Cardiovascular Health Study (CHS) (combined n = 19,611). No SNPs reached genome-wide significance (p<5×10^−8^). SNPs at 6 loci were prioritized for follow-up primarily based on significance of p<10^−4^ and proximity to a known gene (*CSMD2*, *GPR37L1*, *LIN9*, *B4GALNT3*, *GPC5*, and *ZNF592*). The minor allele of *GPC5* (GLYPICAN 5, rs3864180) was associated with a lower risk of SCA in Oregon-SUDS, an effect that was also observed in ARIC/CHS whites (p<0.05) and blacks (p<0.04). In a combined Cox proportional hazards model analysis that adjusted for race, the minor allele exhibited a hazard ratio of 0.85 (95% CI 0.74 to 0.98; p<0.01).

**Conclusions/Significance:**

A novel genetic locus for SCA, *GPC5*, was identified from Oregon-SUDS and successfully validated in the ARIC and CHS cohorts. Three other members of the Glypican family have been previously implicated in human disease, including cardiac conditions. The mechanism of this specific association requires further study.

## Introduction

Sudden cardiac arrest (SCA) claims 250,000–300,000 lives in the US and 4–5 million around the world, on an annual basis [1]. Despite 60 years of significant advancements in rapid emergency response systems and resuscitation methodology in the developed world, the current average rate of survival from SCA in North America is less than five percent [2]. Therefore, the development of effective risk stratification and primary prevention approaches is especially relevant for this significant and global public health issue.

Risk stratification for SCA is an area of active investigation, but available techniques do not identify the vast majority of subjects at risk [3]. Further advancements in this field await the discovery of novel mechanisms related to ventricular arrhythmogenesis. Since two community-based studies confirmed the significant genetic contribution to occurrence of SCA [4], [5], there is increasing recognition of the fact that discovery of novel genetic variants could lead to new mechanistic pathways. The availability of genome-wide genotype data provides the opportunity to conduct a comprehensive and unbiased survey of genetic determinants that could influence the phenotype [6], [7], [8]. Previously, we successfully used a GWAS to identify novel determinants of the QT interval. These variants in *NOS1AP* were subsequently tested and found to be associated with risk for SCA in the general population [9]. An alternative approach is to directly test genetic variants for association with SCA, which is the methodology employed in the current study. The Oregon Sudden Unexpected Death Study (Oregon-SUDS) is an ongoing, prospective, community-based evaluation of approximately one million residents of the Portland, Oregon metropolitan area [10]. Using a case-control approach we performed a GWAS to identify genetic determinants of SCA. Results were validated in two existing large population-based cohorts, the Atherosclerosis Risk in Communities study (ARIC) [11] and the Cardiovascular Health Study (CHS) [12].

## Materials and Methods

This study was conducted according to the principles expressed in the Declaration of Helsinki. The study was approved the Institutional Review Boards of Oregon Health and Science University, Legacy Health Systems, VA Medical Center, Kaiser Permanente NW, Portland OR; Southwest Washington Medical Center, Vancouver, WA; The Johns Hopkins University School of Medicine, Baltimore MD; Wake Forest University, Winston-Salem NC; University of Texas Health Science Center at Houston, TX; University of Washington, Seattle, WA; Group Health, Seattle, WA, USA. Written informed consent was obtained from all enrolled subjects. If subjects were deceased at the time of ascertainment (i.e. following a sudden cardiac death) consent was waived by the respective Institutional Review Boards on grounds of scientific feasibility. These latter subjects were de-identified for the purpose of analysis, in conformation with procedures approved by the respective Institutional Review Boards.

### Overall Study Design

The GWAS was performed on a population-based group of SCA cases and a group of control subjects, both with coronary artery disease (Oregon-SUDS). Additional genotyping of SNPs identified in the GWAS was performed in ARIC and CHS (combined n = 21,680, median follow-up ∼14 yrs).

### Study Populations

#### The Oregon-SUDS

Subjects included in the original GWAS analysis were identified from the ongoing Oregon-SUDS, a population-based study of SCA in the Portland, Oregon metropolitan region (pop. approx. 1,000,000). Methods for this study have been described previously [3], [10], [13]. Briefly, patients with likely out of hospital SCA were referred from the regional emergency medical response system (EMS), the County Medical Examiner, and emergency departments of the 16 area hospitals. SCA was defined as a sudden unexpected pulse-less condition of likely cardiac origin; survivors of SCA were included. If un-witnessed, SCAs were included if subjects were found dead within 24 hours of having last been seen alive and in normal state of health. Subjects were excluded if they had a chronic terminal illness (e.g. terminal cancer), or an identifiable non-cardiac etiology of sudden death related to trauma, overdose, drowning or suicide. For all cases of presumed SCA, three study physicians performed in-house adjudication of SCA cases based on the arrest circumstances, medical records and available autopsy data.

Cases included in the GWAS study were white non-Hispanic SCA cases with DNA available (a blood or tissue sample was available in 59% of cases). Case subjects were also required to have documented significant coronary artery disease (CAD), or, if aged ≥50 years, were assumed to have CAD (based on 95% likelihood of CAD in SCA cases aged ≥50 years) [14], [15]. Documented significant CAD was defined as ≥50% stenosis of a major coronary artery from an angiogram prior to arrest or at autopsy; physician report of past MI; history of percutaneous coronary intervention (PCI) or coronary artery bypass grafting (CABG); autopsy-identified CAD; or MI by clinical data with any two of the following three: ischemic symptoms, positive troponins or CKMB; or convincing pathologic Q waves on ECG.

During the same time period a control group of subjects from the same geographic region were identified who had definite CAD (as defined above) after review of their medical records, but no history of SCA.

### ARIC/CHS

The ARIC study and CHS are population-based prospective cohort studies of cardiovascular disease. The ARIC Study includes 15,792 persons aged 45–64 years at baseline (1987–89), randomly chosen from four US communities [16]. ARIC cohort members completed four clinic examinations, conducted approximately three years apart between 1987 and 1998. CHS includes 5,888 participants >65 years of age identified from four U.S. communities using Medicare eligibility lists. The original cohort included 5201 participants recruited in 1989–1990 and 687 additional subjects were recruited in 1992–1993 to enhance the racial/ethnic diversity of the cohort [17]. Clinic examinations for both ARIC and CHS participants included assessment of cardiovascular risk factors, self-reported medical family history, employment and educational status, diet, physical activity, co-morbidities, and clinical and laboratory measurements. The following exclusion criteria were applied to obtain the final sample for the present analysis: poor quality DNA (samples with <50% of genotypes called), and self-described ethnicity other than White or Black. For ARIC samples, for which a substantial subset has Affymetrix 6.0 genotype data, we excluded samples whose genotypes did not match previous genotype data, genetic outliers identified using EIGENSTRAT [18], and first degree relatives [19]. After these exclusions, 19,611 individuals were available for analysis.

Assessment of SCA has been previously described [9]. Briefly, all cases of fatal CHD that occurred by July 31, 2002 in CHS and December 31, 2002 in ARIC were reviewed and adjudicated by a committee of physicians. SCA was operationally defined as a sudden pulseless condition from a cardiac origin in a previously stable individual, and the reviewers classified each CHD death as definite sudden arrhythmic death, possible sudden arrhythmic death, definite non-sudden death, or unclassifiable. The primary outcome of SCA described in the present study combines both definite and possible sudden arrhythmic death. For the present analysis, participants were censored at time of loss to follow up or death if the cause of death was other than SCA. For 91 cases, SCA status could not be determined.

### GWAS Genotyping and Quality Control

A total of 759 subjects (483 cases, 276 controls) were run on GWAS platforms: 131 cases and 111 controls on the Affymetrix 500K array; 164 cases and 165 controls on the Affymetrix 5.0 array; and 188 cases on the Affymetrix 6.0 array. For the 500K platform, genotypes were called using BRLMM [20], with a quality score setting of 0.3. The 500K platform is run on 2 arrays, each with ∼250,000 SNPs, and we required ≥88% complete data on each array (11 samples failed). For the 5.0 and 6.0 platforms, which genotype ∼500,000 and ∼900,000 SNPs on a single chip, respectively, genotypes were called using Birdseed [21] with a quality score setting of 0.1, and we required ≥95% complete data (1 sample failed). We removed samples for the following criteria: 1) accidental duplicates (4 samples); 2) first degree relatives (1 sample); 3) discordant phenotypic and genotypic sex (9 samples); 4) samples showing too much genetic sharing with all samples (likely contaminated DNA) (4 samples); 5) genetic outliers identified using EIGENSTRAT [18] with a setting of 5 iterations and 6 SD in the top 3 eigenvectors (33 samples). Note that some samples failed more than one QC check. The final dataset consisted of 700 samples (446 cases, 254 controls). We further restricted samples to those ≤80 years of age, to more closely match the cases and controls and potentially enrich for a genetic component to SCA risk, yielding a final dataset of 650 samples (424 cases and 226 controls).

### Merging GWAS Data and Imputation

Monomorphic SNPs and SNPs with no genotypes calls (100% missing) were removed from each dataset (500K, 5.0, 6.0). First, the 500K and 5.0 datasets were merged, and subsequently all SNPs with >10% missing data and/or Hardy-Weinberg equilibrium (HWE) P<0.00001 were removed from the 500K/5.0 dataset (final n = 436,267 SNPs), and the 6.0 dataset (final n = 902,958 SNPs), and these 2 datasets were merged. Imputation to fill in missing data was performed on the combined dataset using MACH v1.16, with 50 rounds and 200 states [22]. SNPs that were significantly different between cases genotyped on the 500K/5.0 platforms and 6.0 platforms (n = 14) and SNPs with minor allele frequency <0.01 were removed, and an additional round of imputation was performed using the HapMap CEU dataset (r21a) as a reference to generate genotype data on all Phase II HapMap SNPs (n = 2,557,332).

### Quality Control for GWAS SNPs

Using QQ plots to identify classes of SNPs showing inflated results, and thus a high likelihood of false-positives, we excluded SNPs with low imputation quality (rsq <0.85), MAF <0.02, HWE >0.001, and any SNP flagged by MACH as having a significantly different allele frequency between the dataset and the reference HapMap data. These criteria yielded 1,966,233 SNPs for analysis with a genomic control inflation factor of 1.015, showing no evidence for population stratification or inflated results due to imputation.

### Validation Genotyping

Genotyping was performed using iPlex single base primer extension with MALDI-TOF mass spectrometry according to manufacturer protocols (Sequenom Inc., San Diego, CA). PCR and extension primer sequences are available upon request.

### Statistical Analyses

Analyses of the GWAS data were performed in PLINK [23], using logistic regression adjusting for age and sex under an additive genetic model. In ARIC/CHS, all analyses were stratified by self-reported ethnicity. Because the study protocols were similar across ARIC and CHS, the number of SCA events was modest within each study, and initial genotype-phenotype analyses indicated that the associations did not differ between the two studies, all analyses were pooled across the two studies to increase statistical power. The rare allele of each SNP in whites was designated as the minor allele. Deviations from Hardy-Weinberg proportions were assessed using the chi-squared goodness of fit test within each ethnicity group. For SCA risk, single SNP genotype-based analyses were performed. To estimate the relative hazards and the significance of the association between each SNP genotype and SCA risk while adjusting for covariates, Cox proportional hazards models were constructed, and a Bonferroni-corrected-alpha of 0.0125 (0.05/4) was used to declare statistical significance in the age, sex, and study adjusted analysis. An additive model was assumed for each SNP. For significant SNPs, a model assuming 3 genotypic risks was also constructed to confirm the use of the additive model. The proportional hazards assumption was checked with Schoenfeld's residual [24]. As exploratory analyses, the role of genotypic effects across various high-risk subgroups was examined both with stratified analyses and by fitting interaction terms into the regression models. Analyses were performed in R (version 2.6.2).

## Results

### Baseline characteristics of subjects in the Oregon-SUDS

Case (n = 424) and control (n = 226) subjects in the Oregon-SUDS GWAS were both predominantly male (72.9% vs. 68.6%, p = 0.25), while case subjects were younger than controls (59.4±12.3 vs. 63.2±9.9 yrs, p<0.0001); 75% of cases and 90% of controls were age 50 or older (p<0.0001). All subjects included in the Oregon-SUDS GWAS were white, based on inclusion criteria. Risk factors for heart disease were common among both cases and controls (diabetes mellitus was diagnosed in 29% cases and 34% controls, p = 0.25; obesity in 45% cases vs. 42% controls, p = 0.41. Among all SCD cases age ≥50, 58% of women and 63% of men had documented significant CAD. Fifteen percent of cases age ≥50 had an autopsy performed; in this subset, 85% of women and 86% of men had significant CAD noted at autopsy. While a history of prior cardiac events was relatively common among case subjects (22% with prior MI; 22% with prior revascularization), a history of prior cardiac arrest was uncommon (4 case subjects; 1% of case population).

### Oregon-SUDS GWAS

**Figure 1:**
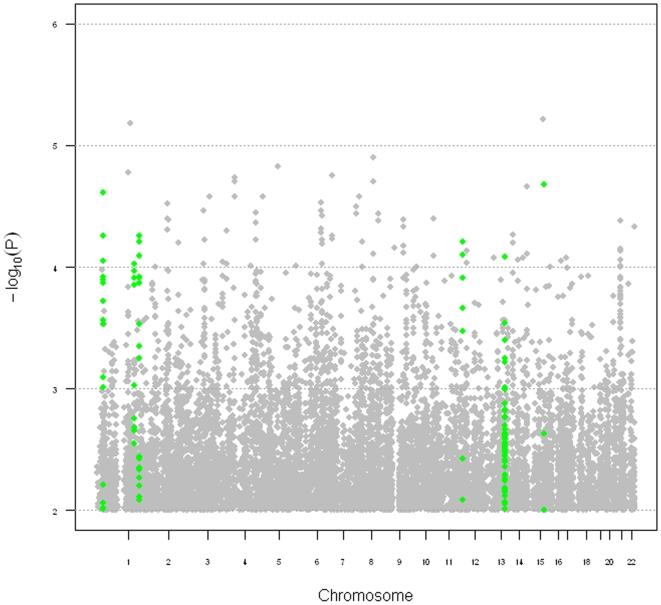
Summary of GWAS results from the Oregon-SUDS population. Chromosome is on the X-axis and the negative log of the P value is on the Y-axis. Regions meeting criteria for follow-up in ARIC/CHS are highlighted in green.

Since Oregon-SUDS samples were assayed across multiple Affymetrix platforms, we performed several rounds of data cleaning (of both samples and SNPs) as well as imputation of non-genotyped SNPs, ultimately yielding 650 samples and ∼2 million SNPs for analysis (see Methods). We performed a logistic regression, adjusting for age and sex, and the genomic control factor (λ) was 1.002, showing no evidence for population stratification or inflated results due to imputation. No SNPs met genome-wide significance (P<5×10^−8^, Figure 1, Table 1), and thus we prioritized SNPs for follow-up based on the following criteria: 1) significance (p<10^−4^); 2) multiple adjacent SNPs showing similar levels of association (to eliminate associations due to genotyping artifacts); 3) within 25 kb of a known gene; and 4) minor allele frequencies among controls not significantly different (P<0.01) when compared to additional controls obtained from the Wellcome Trust Case Control Consortium (WTCCC) data. This final criterion was implemented to remove SNPs for which associations appeared to be driven by the controls rather than the cases, suggesting that imputation was of poor quality.

**Table 1:** Top results from the Oregon-SUDS GWAS.

SNP	CHR	Position	MAF	Rsq	OR	P	GENE	P*
rs16835611	1	33,710,878	0.18	1.0000	0.59	9.00E-05	CSMD2	0.02
rs7516762	1	198,824,237	0.11	0.9983	2.35	9.38E-05	GPR37L1	0.34
rs2306119	1	222,727,457	0.39	0.9952	0.61	5.60E-05	LIN9	0.08
rs7313849	12	481,855	0.09	0.8512	0.44	6.25E-05	B4GALNT3	0.15
rs3864180	13	91,234,489	0.39	0.9723	0.61	8.20E-05	GPC5	0.06
rs1429445	15	83,085,569	0.09	0.9335	3.08	2.11E-05	ZNF592	0.24

CHR = chromosome; MAF = minor allele frequency; Rsq = measure of imputation quality from MACH [22]; OR = odds ratio;

P* = P value comparing Oregon-SUDS controls to controls from the Wellcome Trust Case Control Consortium.

Six SNPs met these criteria (Table 1, Figure 1), and were selected for validation in the combined ARIC and CHS cohorts.

### Validation in ARIC/CHS

Of the six candidate SNPs from the Oregon-SUDS GWAS, one failed assay design, and 1 was out of Hardy Weinberg equilibrium (HWE) (P<0.001) in both whites and blacks from the ARIC/CHS cohort. This SNP was also missing >5% data, suggesting poor genotyping, and thus was subsequently dropped from all analyses. Of the remaining 4 SNPs, all were in HWE (P>0.01) in whites and blacks, with the exception of rs7313849 in blacks (P<0.0004), and all had <5% missing data.

**Figure 2:**
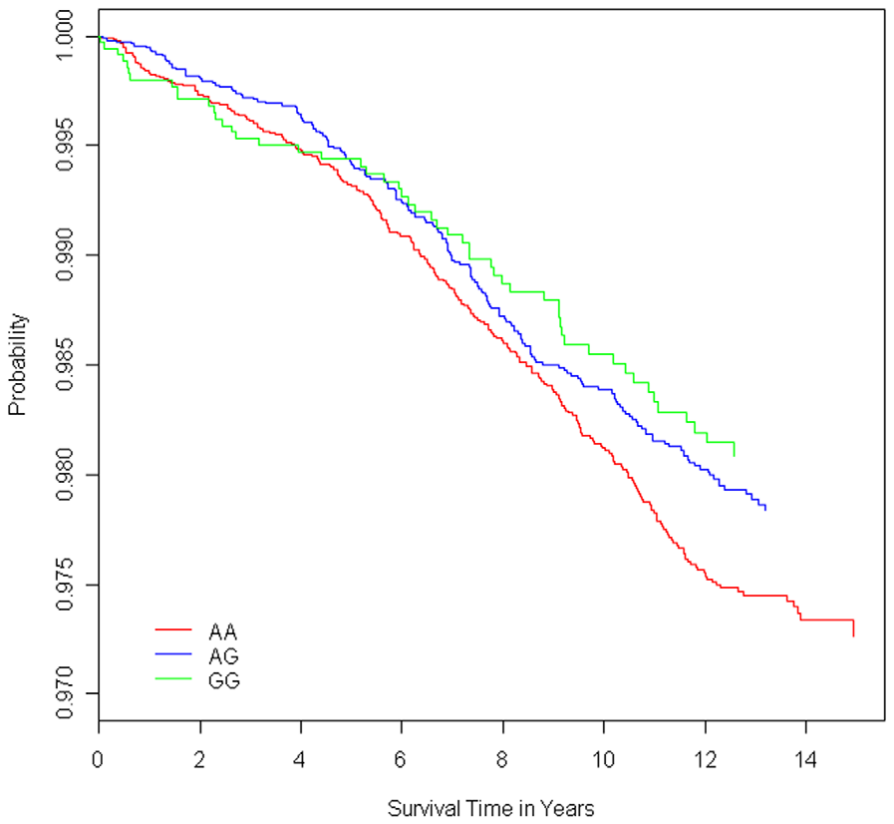
Survival curves (free of sudden cardiac arrest) stratified by rs3864180 genotype in the combined ARIC/CHS cohort. Cox proportional hazards model was adjusted for age, sex, and race/ethnicity. Individuals homozygous for the protective allele (GG) are shown in green, heterozygotes (AG) in blue, and homozygous for the risk allele (AA) are in red.

In our final combined ARIC/CHS dataset after data cleaning (see Methods), 521/19,520 individuals experienced SCA over a median follow-up time of 13.5 years (14.1 in ARIC and 12.2 in CHS). We performed Cox proportional hazards models for each SNP, adjusting for age and sex, with time to SCA or loss to follow-up as the outcome variable. Only rs3864180, an intronic SNP, was significantly associated with SCA, and this association was observed in both whites (P<0.05) and blacks (P<0.039) (Table 2). In a combined analysis of both whites and blacks, we observed a relative risk of 0.85 (95% CI, 0.74–0.98, P<0.01), after adjusting for age, sex, and race/ethnicity (Figure 2). Additional analyses were conducted to examine the impact of other CVD risk factors on the association of rs3864180 with SCA. In the combined racial cohort, we observed a statistically significant stronger effect in ARIC (0.78) vs. CHS (0.94), as well as a stronger effect in women (0.72) than in men (0.94) (Table S1). No other risk factors showed a statistically significant interaction (P<0.05) with the SNP, though we did observe stronger effects for those without previous MI, age younger than 70 years, and the absence of diabetes at baseline.

**Table 2:** Validation of top GWAS hits in combined ARIC and CHS cohorts, stratified by race/ethnicity.

				Whites				Blacks			
SNP	CHR	Position	GENE	MAF	RR	SE	P	MAF	RR	SE	P
rs16835611	1	33,710,878	CSMD2	0.16	0.93	0.11	0.25	0.03	0.94	0.31	0.42
rs7313849	12	481,855	B4GALNT3	0.12	0.96	0.11	0.37	0.43	0.99	0.11	0.48
**rs3864180**	**13**	**91,234,489**	**GPC5**	**0.40**	**0.88**	**0.08**	**0.05**	**0.15**	**0.74**	**0.17**	**0.039**
rs1429445	15	83,085,569	ZNF592	0.20	0.94	0.10	NS	0.05	1.03	0.24	0.45

NS indicates that the effect is in the opposite direction of Oregon-SUDS, P-values are one-sided. CHR = chromosome; MAF = minor allele frequency; RR = relative risk; SE = standard error. **Bold** indicates significant association with SCA.

### Association of rs3864180 with QT interval

We performed linear regression analysis on QT interval adjusted for age, sex, heart rate, race/ethnicity and study site (Table 3) and observed a decrease of 0.47±0.18 ms in the QT interval with the allele associated with reduced risk of SCA (P<0.012). Further, we saw a statistically significant interaction between rs3864180 and sex (P<0.012), with a stronger effect in women, which was also seen in the association with SCA; although not statistically significant, the effect on QT interval was observed separately for both ARIC and CHS, and for both blacks and whites. No significant difference was observed between ARIC and CHS samples. Exclusion of all SCA cases did not significantly change these results (data not shown).

**Table 3:** Effect of rs3864180 on QT interval in ARIC/CHS.

Cohort	N	Beta	SE	P value	P*
**All**	**18,152**	**−0.47**	**0.18**	**0.012**	
ARIC	13,587	−0.36	0.21	0.09	NS
CHS	4,561	−0.67	0.39	0.08	
Blacks	4,229	−1.04	0.57	0.07	NS
Whites	13,919	−0.36	0.19	0.06	
**Women**	**10,306**	**−0.72**	**0.27**	**0.007**	**0.012**
Men	7,839	−0.09	0.25	0.71	

Beta is in ms. SE = standard error;

P* is the P value for the interaction term. **Bold** indicates significant association for QT interval.

## Discussion

In this case-control study, we found that the minor allele of rs3864180 (SNP in GPC5) conferred protection against SCA among subjects recruited in the Oregon-SUDS. Furthermore, this effect was validated among white and black subjects recruited in the ARIC and CHS cohorts. In a Cox proportional hazards model combining whites and blacks, adjusted for baseline age, sex, and race, the risk ratio for the minor allele was 0.85 (95% CI 0.74 to 0.98; p<0.01).

Given that SCA is known to have a genetic component, the identification of genetic variants that modulate risk can contribute to an improved understanding of mechanisms. CAD is the disease condition most commonly associated with SCA and is observed in at least 80% of overall cases. Hence, our use of a population based design in which all subjects had CAD increased the likelihood that we would identify genetic variants that contribute specifically to SCA among subjects with existing CAD. Prolongation of the QT interval has been associated with increased SCA risk in the community [13]. It is of interest that rs3864180 was also associated with a small but significant effect on duration of the corrected QT interval, with the protective allele associated with shorter QT interval (P<0.012). For both SCA and QT interval, we observed a significantly stronger effect in women. While these trends were not significant, risk of SCA was higher in those without history of previous MI, younger than 70 years of age, and no diabetes at baseline. These findings are consistent with the expectation of observing stronger genetic effects in individuals with fewer co-morbidities. Since rs3864180 is a non-coding SNP, the possibility exists that it is either in linkage disequilibrium with a coding SNP or has a regulatory function.

The GPC5 gene encodes glypican 5, a member of the heparan sulfate preoteoglycans (HSPGs) that are bound to the external surface of the plasma membrane by glycosyl-phosphatidylinositol (GPI) linkage [25]. There are six glypican family members in the Human Genome (GPC1 to GPC6). While defects in GPC5 have yet not been reported to be associated with disease, mutations in three other members of the family have been associated with two distinct syndromes. The Simpson-Golabi-Behmel syndrome (SGBS) is a X-linked overgrowth/malformation syndrome caused by mutations in GPC3 and GPC4 [26], [27]. Cardiac arrhythmias have been reported in patients with SGBS, likely contributing to high incidence of death in early infancy and cardiac arrest in the adult [28]. Also, autosomal recessive omodysplasia is a genetic condition characterized by severe short stature and congenital heart defects, caused by homozygosity for null mutations in GPC6 [29].

In the cardiovascular system, cell surfaces and surrounding extracellular matrix express large quantities of HSPGs. These are involved in regulating vasculogenesis and angiogenesis after ischemic injury, interactions of cells with adhesive proteins and blood vessels, proliferation of smooth muscle cells during atherogenesis, metabolism of lipoproteins and non-thrombogenic characteristics of endothelial cells [30]. A functional evaluation of rs3864180 and other variations in the GPC5 gene is warranted. There are several interesting possibilities for a role in the pathobiology of SCA.

Several limitations should be considered with regard to interpretation of our results. First, it is not known whether we have identified the functional variant, or just a marker in LD with the functional variant. Second, while rs3864180 is found within an intron of *GPC5*, this does not definitively prove the involvement of *GPC5* in SCD risk, since it is possible for the functional variant to act upon a more distant gene [31]. Finally, appropriate caution should be exercised in extrapolating these results to other populations. It should be recognized that rs3864180 did not reach genome-wide significance (P<5×10^−8^) in either the original GWAS or the validation study. Thus while validation of the original GWAS finding in an independent population provides evidence for a true association with lower SCD risk, additional replication of these findings is warranted.

### Conclusion

A novel, protective genetic locus for SCA, GPC5, was identified from a large case-control study (Oregon-SUDS) and successfully validated in the ARIC and CHS cohorts. The mechanism of this association requires further study.

## Supporting Information

Table S1 Protective effect of rs3864180 on risk of SCA in ARIC/CHS stratified by risk factors for cardiovascular disease.
(0.11 MB DOC)
