# Supplementary material for: Genome-Wide Association Study Identifies GPC5 as a Novel Genetic Locus Protective against Sudden Cardiac Arrest
Source: PLoS One. 2010 Mar 25;5(3):e9879. doi: 10.1371/journal.pone.0009879 (PMC2845611; doi:10.1371/journal.pone.0009879)
Supplement: Table S1 — Protective effect of rs3864180 on risk of SCA in ARIC/CHS stratified by risk factors for cardiovascular disease. (0.11 MB DOC) [file pone.0009879.s001.doc]

|  | **Whites** | | | | | **Blacks** | | | | | **Combined** | | | | |
| --- | --- | --- | --- | --- | --- | --- | --- | --- | --- | --- | --- | --- | --- | --- | --- |
|  | **N** | **RR** | **95% CI** | **P** | **P*** | **N** | **RR** | **95% CI** | **P** | **P*** | **N** | **RR** | **95% CI** | **P** | **P*** |
| ARIC | 10,435 | 0.84 | 0.67-1.05 | 0.06 | NS | 3,777 | 0.63 | 0.41-0.96 | 0.015 | NS | **14,212** | **0.78** | **0.64-0.95** | **0.007** | **0.049** |
| CHS | 4,332 | 0.92 | 0.75-1.13 | 0.21 |  | 775 | 1.08 | 0.62-1.89 | NA |  | **5,107** | **0.94** | **0.77-1.14** | **0.26** |  |
|  |  |  |  |  |  |  |  |  |  |  |  |  |  |  |  |
| No History of MI | 13,907 | 0.84 | 0.70-1.00 | 0.025 | NS | 4,347 | 0.68 | 0.46-1.01 | 0.025 | NS | 18,254 | 0.80 | 0.68-0.95 | 0.004 | NS |
| History of MI | 860 | 1.00 | 0.76-1.31 | 0.49 |  | 198 | 0.91 | 0.48-1.72 | 0.39 |  | 1,058 | 0.98 | 0.76-1.26 | 0.44 |  |
|  |  |  |  |  |  |  |  |  |  |  |  |  |  |  |  |
| Women | **7,957** | **0.70** | **0.53-0.92** | **0.005** | **0.045** | 2,823 | 0.78 | 0.49-1.24 | 0.15 | NS | **10,780** | **0.72** | **0.57-0.91** | **0.003** | **0.026** |
| Men | **6,810** | **0.98** | **0.82-1.18** | **0.42** |  | 1,729 | 0.70 | 0.43-1.13 | 0.08 |  | **8,539** | **0.94** | **0.79-1.11** | **0.22** |  |
|  |  |  |  |  |  |  |  |  |  |  |  |  |  |  |  |
| Age <70 | 6,459 | 0.93 | 0.73-1.18 | 0.27 | NS | **2,671** | **0.50** | **0.30-0.81** | **0.003** | **0.006** | 9,130 | 0.80 | 0.65-0.99 | 0.018 | NS |
| Age ≥70 | 8,308 | 0.86 | 0.71-1.04 | 0.08 |  | **1,881** | **1.29** | **0.81-2.05** | **NA** |  | 10,189 | 0.91 | 0.86-1.09 | 0.15 |  |
|  |  |  |  |  |  |  |  |  |  |  |  |  |  |  |  |
| No Diabetes | 13,197 | 0.84 | 0.71-1.01 | 0.03 | NS | 3,619 | 0.71 | 0.45-1.12 | 0.07 | NS | 16,816 | 0.82 | 0.70-0.97 | 0.010 | NS |
| Diabetes | 1,554 | 0.97 | 0.72-1.29 | 0.40 |  | 903 | 0.80 | 0.48-1.33 | 0.19 |  | 2,457 | 0.92 | 0.71-1.18 | 0.25 |  |
|  |  |  |  |  |  |  |  |  |  |  |  |  |  |  |  |
| No Hypertension | 9,521 | 0.89 | 0.71-1.13 | 0.18 | NS | 1,900 | 0.78 | 0.36-1.70 | 0.27 | NS | 11,421 | 0.88 | 0.70-1.10 | 0.14 | NS |
| Hypertension | 5,241 | 0.87 | 0.71-1.06 | 0.08 |  | 2,650 | 0.75 | 0.52-1.09 | 0.07 |  | 7,891 | 0.84 | 0.70-0.99 | 0.020 |  |
|  |  |  |  |  |  |  |  |  |  |  |  |  |  |  |  |
| BMI <30 kg/m2 | 11643 | 0.89 | 0.75-1.06 | 0.10 | NS | 2769 | 0.6 | 0.39-0.93 | 0.012 | NS | 14412 | 0.84 | 0.71-0.99 | 0.016 | NS |
| BMI ≥30 kg/m2 | 3103 | 0.87 | 0.65-1.16 | 0.18 |  | 1772 | 1.04 | 0.62-1.75 | NA |  | 4875 | 0.9 | 0.70-1.17 | 0.22 |  |
|  |  |  |  |  |  |  |  |  |  |  |  |  |  |  |  |
| HDL <40 mg/dL | 3820 | 0.98 | 0.78-1.24 | 0.44 | NS | 736 | 0.62 | 0.28-1.35 | 0.12 | NS | 4556 | 0.94 | 0.75-1.17 | 0.29 | NS |
| HDL ≥40 mg/dL | 10918 | 0.82 | 0.67-1.00 | 0.024 |  | 3658 | 0.75 | 0.52-1.09 | 0.07 |  | 14576 | 0.8 | 0.67-0.96 | 0.007 |  |
|  |  |  |  |  |  |  |  |  |  |  |  |  |  |  |  |
| LDL <130 mg/dL | 6788 | 0.81 | 0.64-1.03 | 0.04 | NS | **2071** | **1.00** | **0.62-1.60** | **0.50** | **0.024** | 8859 | 0.84 | 0.68-1.04 | 0.06 | NS |
| LDL ≥130 mg/dL | 7726 | 0.94 | 0.76-1.14 | 0.26 |  | **2286** | **0.43** | **0.24-0.76** | **0.002** |  | 10012 | 0.83 | 0.69-1.00 | 0.025 |  |

RR = relative risk; CI = confidence interval; NS = not significant. NA indicates that the effect is in the opposite direction from that observed in Ore-SUDS. P values are one-sided for the protective effect of rs3864180 on SCA. P* is the significance of the association of the interaction term (two-sided P value). **Bold** indicates significant interaction terms.
